# Spasticity in Prolonged Disorders of Consciousness: A Prospective Cohort Study

**DOI:** 10.3390/brainsci16050524

**Published:** 2026-05-14

**Authors:** Nathalie Draulans, Cecile Utens, Danielle Driessen, Willemijn van Erp, Gerard Ribbers, Jörg Wissel, Aurore Thibaut

**Affiliations:** 1 Libra Rehabilitation & Audiology, P.O. Box 5022, 5004 EA Tilburg, The Netherlands; n.draulans@libranet.nl (N.D.); d.driessen@libranet.nl (D.D.); willemijn.vanerp@radboudumc.nl (W.v.E.); 2 Department of Rehabilitation Medicine, Erasmus Medical Centre, P.O. Box 2040, 3000 CA Rotterdam, The Netherlands; g.ribbers@erasmusmc.nl; 3 Department of Primary and Community Care, Radboud University Medical Centre, Postbus 9101, 6500 HB Nijmegen, The Netherlands; 4 Neurology and Psychosomatic at Wittenbergplatz, University of Potsdam, Ansbacher Street 17-19, 10787 Berlin, Germany; joerg@schwarz-wissel.de; 5 Coma Science Group, GIGA-Consciousness, University of Liege, Avenue de l’hôpital 11, 4000 Liège, Belgium; athibaut@uliege.be

**Keywords:** spasticity, severe brain injury, disorders of consciousness, neurorehabilitation

## Abstract

**Highlights:**

**What are the main findings?**

- The prevalence of spasticity in patients with prolonged disorders of consciousness is high and widespread with bilateral involvement in almost all patients. Severe spasticity is present in 19 to 30% of patients.
- Prevalence of spasticity and severity of spasticity increased over the treatment period.
- There was no association between spasticity and level of consciousness or recovery of consciousness. An association between pain/discomfort and spasticity was found, without establishing the direction of the relationship.

**What are the implications of the main findings?**

- Spasticity should be anticipated rather than considered a secondary complication, and treated as a distinct clinical problem.
- Findings emphasize the need for prospective interventional studies to optimize pharmacological and non-pharmacological spasticity management in patients with prolonged disorders of consciousness.

**Abstract:**

**Background:** Spasticity is a frequent and disabling complication in patients with prolonged disorders of consciousness (PDOC), yet its prevalence, distribution, evolution, and relationship with recovery of consciousness remain poorly characterized. The aim was to investigate the prevalence, severity, distribution, and evolution of spasticity in PDOC patients undergoing early intensive neurorehabilitation (EIN), and to explore clinical factors associated with spasticity and its relationship with level of consciousness (LOC). **Methods:** This study was embedded in the nationwide prospective DOCTOR cohort and included 126 PDOC patients admitted for EIN in the Netherlands between 2019 and 2023. Spasticity was assessed at admission and discharge using the Ashworth Scale (AS) across seven bilateral muscle groups. Associations between spasticity, demographic and clinical variables, medication use, nociception, and recovery of consciousness were analyzed. **Results:** Spasticity was highly prevalent at EIN admission (88%) and discharge (90%), with mostly bilateral and widespread involvement. Elbow flexors, wrist flexors, hip adductors, and knee flexors were most frequently affected. Severe spasticity was present in 19% at admission and 30% at discharge. Spasticity severity correlated positively with pain scores and use of spasmolytics, but not with LOC. No association was found between spasticity at admission and recovery of consciousness. **Conclusions:** Spasticity is nearly ubiquitous and often progressive in PDOC, even during specialized neurorehabilitation. Its evolution appears independent of recovery of consciousness, underscoring the need to assess and manage spasticity as a distinct clinical entity. Prospective interventional studies are warranted to optimize spasticity treatment in this population.

## 1. Introduction

Patients with disorders of consciousness (DOC) due to severe brain injury exhibit no or extremely limited signs of awareness and have significant physical disability. When such state has lasted for more than 28 days, the patient can be diagnosed as prolonged disorders of consciousness (PDOC) [1]. PDOC includes the Unresponsive Wakefulness Syndrome (UWS) also known as vegetative state, where patients display reflexive movements and eye opening but lack overt awareness of themselves or their surroundings, and the Minimally Conscious State (MCS), in which patients exhibit fluctuating purposeful behaviors but are unable to communicate or use objects functionally [2,3]. MCS is further subcategorized in MCS− and MCS+ based on, respectively, the absence or presence of language-related behaviors [4]. Once either functional communication or functional object use is present, the diagnosis of emergence from MCS is established. Signs of consciousness are usually detected by evaluating behavioral responses, with the most widely recommended tool being the Coma Recovery Scale-Revised (CRS-R) [5].

Spasticity is often reported as a major complication in patients with PDOC and a major cause of motor disabilities. Reported prevalence of spasticity ranges from 21% to 100% [6,7,8,9]. However, these studies provide no other information besides whether spasticity is present or not.

It has been hypothesized that recovery of consciousness may be associated with increased spasticity, potentially due to enhanced processing and responsiveness to nociceptive stimuli such as pain [10,11]. Nociceptive processing in PDOC is often intact and increased nociceptive input may exacerbate spasticity [11,12]. However, the direction of this relationship has not been directly established. Conversely, spasticity may improve with recovery of consciousness, as both may reflect parallel recovery processes of the underlying neurological dysfunction rather than independent phenomena [13,14]. However, evidence supporting these different hypotheses remains limited.

Spasticity may also impact the evaluation of consciousness through its negative impact on motor responses, cause significant discomfort and increase caregiver burden [11,15]. Furthermore, spasticity and paresis are linked to changes in musculotendinous properties, also known as spastic myopathy, which lead to restrictions in both passive and active movement, especially at chronic stages [16,17].

Despite its major impact and the several hypotheses that have been formulated, only a few studies focused on spasticity in PDOC. Few articles address the severity, distribution, and evolution of spasticity [11,18,19,20], measuring spasticity with the Modified Ashworth Scale (MAS) or Ashworth Scale (AS) [14]. The number of patients included varied between 19 and 210, and all studies were performed retrospectively. Only two of these studies [18,19] were longitudinal, providing information on the evolution of spasticity. Finally, only one paper identified possible factors associated with the development of spasticity over time in 19 patients [19]; these were etiology (patients with TBI were less likely to develop spasticity in the upper limbs) and level of consciousness (patients with lower levels of consciousness at baseline were more likely to develop spasticity in the lower limbs).

The start of a novel, nationwide chain of care for PDOC patients in the Netherlands in 2019 sets an ideal setting for prospective research with a very low inclusion bias as virtually all PDOC patients are centrally referred to one center for 14 weeks of Early Intensive Neurorehabilitation [21,22]. The current study is an exploratory investigation using data from a large prospective cohort study, i.e., the DOCTOR study [22]. It examines prevalence of spasticity in patients with PDOC with the AS, its severity, distribution among muscle groups, and evolution of spasticity in individuals. Furthermore, this study explores factors correlated with the presence and progression of spasticity and explores whether the data support any of the given hypotheses on the relation between the evolution of spasticity and the evolution of LOC.

## 2. Materials and Methods

### 2.1. Design, Setting, and Participants

The current study is embedded in the DOCTOR study (Disorders Of Consciousness, Treatment and Outcomes Registry), a prospective cohort study on the outcomes of early intensive neurorehabilitation (EIN) for PDOC with a 2-year follow-up [22].

When individuals with PDOC were admitted for EIN, they could be included in the DOCTOR study if they were 16 years or older, had a first brain injury, and had PDOC lasting more than 4 weeks but less than 6 months at the time of admission as diagnosed with the Coma Recovery Scale-Revised (CRS-R). Additional eligibility criteria for admission to EIN are weaned from a ventilator, medical stability (e.g., no oxygen suppletion, no intravenous antibiotic treatment), and absence of uncontrollable epilepsy. Inclusion for the DOCTOR study was performed between April 2019 and April 2023.

### 2.2. Early Intensive Neurorehabilitation

EIN (described in detail by Driessen et al. [22]) is an interdisciplinary program of 14 weeks maximally, with a team of rehabilitation physicians, physiotherapists, occupational therapists, speech therapists, neuropsychologist, cognitive rehabilitation therapists, and nursing staff offering an integrated care program. EIN is delivered in the post-acute phase after injury at the inpatient ward of a rehabilitation center. Nursing care is 24/7 available and out of office hours medical doctors are on call available. In the case of unexpected medical episodes that require acute specialized care, this is available from an adjacent hospital. EIN includes a minimum duration of 120 min therapy per day on weekdays, divided over a maximum of 5 therapy sessions per day. EIN focuses on optimizing the conditions for recovery of consciousness, regaining motor abilities and cognitive functioning in the process of recovery. For spasticity, the program includes physiotherapy and occupational therapy interventions, such as stretching, positioning and/or splinting, and/or pharmacological therapy like the administration of oral or intramuscular spasmolytics [22]. Patients who regain consciousness before 14 weeks are discharged earlier to an appropriate rehabilitation setting or other treatment facility. Patients who remain in PDOC at the end of EIN are eligible for prolonged intensive neurorehabilitation in 1 of 3 specialized nursing homes, up until 2 years post-injury [21].

### 2.3. Data Collection

Spasticity was measured at EIN admission and discharge. EIN is 14 weeks but shorter in the case of regaining consciousness before the 14th week. To assure comparability of the discharge data, only measurements performed ±2 weeks of the discharge date were used.

### 2.4. Outcome Measures

Spasticity was measured with the AS, which is an ordinal scale with five levels, where higher scores indicate more severe muscle hyper resistance or muscle stiffness to passive stretch [14]. Although we recognize that the AS measures perceived resistance, which is a combination of neurological and rheological factors, similar to other studies in PDOC populations, we will refer to the result of the AS measurement as the degree of spasticity [11,19,20,23]. All AS assessments were performed by two trained physiotherapists together [24] with extensive experience in working with patients in PDOC. The inter- and intra-rater reliability of the AS have been demonstrated to be moderate to good (Cohen’s kappa >0.40 and 86% agreement) [24,25].The following muscle groups were assessed bilaterally: elbow flexors, elbow extensors, wrist flexors, hip adductors, knee extensors, knee flexors, and plantar flexors (with extended knee). In total, 14 measurements per patient per time point were performed (6 in the upper limb and 8 in the lower limb). An AS score of ≥1 in one of the tested muscle groups was considered as presence of spasticity. AS scores ≥3 in one of the tested muscle groups was considered as the presence of severe spasticity. We calculated summed AS scores, as well the highest AS value within the patient to express overall spasticity and severity of spasticity.

### 2.5. Clinical and Demographic Characteristics

Level of consciousness was measured with the CRS-R. The CRS-R is a bedside assessment tool for differentiating levels of consciousness (UWS, MCS−, and MCS+) and emerged from MCS (EMCS) by observation of responses to various stimuli [5]. It is composed of 23 items on 6 hierarchical subscales as follows: auditory, visual, motor, oromotor/verbal, communication, and arousal, leading to a total score varying between 0 and 23 and domain scores. Pain/discomfort was measured with the Nociception Coma Scale-Revised (NCS-R), at rest, during the CRS-R assessment. NCS-R leads to a total score varying in the range of 0–12, with higher scores indicating higher levels of possible discomfort or pain [26,27]. CRS-R and NCS-R were assessed weekly, but only scores at admission and discharge were used in the current study. Demographic characteristics, etiology (i.e., TBI or non-TBI), time since injury (TSI), use of spasmolytics, analgesics and botulinum injections, and length of stay (LoS) in the EIN program were obtained from the medical records.

### 2.6. Statistical Analysis

Descriptive statistics were used to describe clinical and demographic characteristics at admission, the presence of spasticity, distribution and severity of spasticity, evolution of spasticity, and use of medication. Baseline comparison of patients who were discharged before the end of the 14-week EIN program and those who received the full 14 weeks were performed using Mann–Whitney tests in the case of continuous variables and chi-squared tests in the case of categorical variables.

Patients with ≥7 valid AS measurements per time point were included in the calculations of presence of spasticity, summed AS scores, and the highest AS score. For the calculation of upper limb spasticity (i.e., AS score ≥1 in one of the muscle groups in the upper limb) ≥3 measurements per time point were required. For spasticity in the lower limb (i.e., spasticity in one of the muscle groups in the lower limbs), ≥4 measurements were required. Spasticity in all four limbs was defined as an AS score ≥1 in one of the muscle groups belonging to the left and right upper limb and left and right lower limb. Evolution of AS scores was analyzed using the summed AS scores and highest AS scores.

Differences between admission and discharge in the proportion of patients having (severe) spasticity and the proportion of patients using medication were analyzed using McNemar tests. Differences in AS sum scores between admission and discharge were analyzed using Wilcoxon signed rank test.

Correlation between the presence of spasticity and clinical and demographic characteristics both at admission and discharge was analyzed using univariate logistic regression. Presence of spasticity yes/no at either admission or discharge was the dependent variable. Age, TSI, type of injury, gender, CRS-R total score, CRS-R motor sub score, use of medication at admission, and NCS-R score were independent variables for the analysis of spasticity at admission. For the analysis at discharge, CRS-R total score at discharge, CRS-R motor sub score at discharge and NCS-R score at discharge, length of stay, use medication at discharge and presence of spasticity at admission were independent variables. Results are presented as odds ratios (OR) with 95% confidence intervals (CI).

Correlations between AS sum score or difference in AS sum score and demographic and clinical factors were analyzed using Pearson’s r correlation analysis. Same demographic and clinical factors were analyzed as for the analysis of presence of spasticity, extended with the following factors: difference in CRS-R total score, CRS-R motor score, and NCS score for the difference in AS sum score analysis. Correlations between evolution of level of consciousness and spasticity were analyzed using the appropriate test, depending on the type of variable, i.e., chi-squared test, Pearson correlation test, or independent *t*-test.

Given the exploratory nature of the study and the use of multiple statistical approaches addressing different research questions, no formal correction for multiple testing was applied.

All analyses were performed using SPSS 28 and R 4.5.1 and RStudio 2026.01.0. A significance level of ≤0.05 was considered significant.

### 2.7. Ethical Considerations

Written informed consent for participation in the DOCTOR study was obtained from the legal representative of each patient before the start of the study. This study was determined by the Medical Ethics Committee of Erasmus MC, University Medical Centre Rotterdam, not to be subject to the “Medical Research Involving Human Subjects Act” (MEC-2019-0127).

## 3. Results

### 3.1. Participants

**Figure 1:**
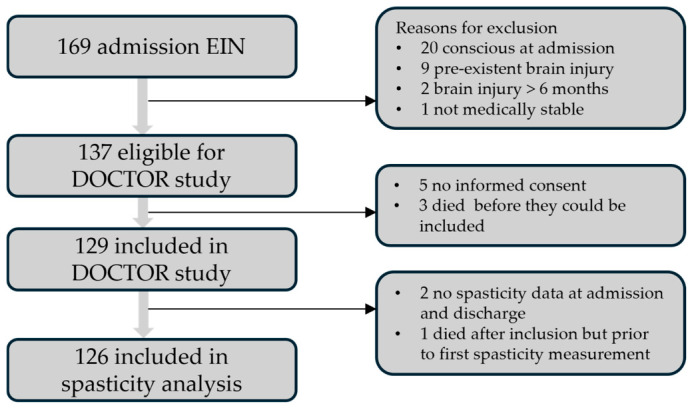
Flowchart. DOCTOR: Disorders Of Consciousness Treatment and Outcomes Registry; EIN: early intensive neurorehabilitation.

In the DOCTOR study, 169 patients were screened for eligibility, of whom 129 were included. For our study, data of 126 patients were available. Two patients had no spasticity data at start EIN nor at discharge from EIN. One patient died in the first week of EIN, having no spasticity data. See Figure 1 for the flowchart through the study.

Demographic and clinical characteristics of the 126 patients included are presented in Table 1. Mean (SD) age was 38.3 years (16.4), 52% were men, and 56% had a traumatic etiology. Fifty-five (44%) patients regained consciousness during EIN [27]. Six patients died during EIN, but one after the planned AS measurement at 14 weeks. Average length of EIN was 94 days (13–14 weeks), but 50 patients (40%) were discharged earlier, mostly because they regained consciousness. These patients had significantly shorter TSI and significantly better LOC at admission.

Valid measurements (i.e., ≥7 valid AS measurements for the different muscle groups) were available for 117 patients at admission and 104 patients at discharge. Measurements were missing for different reasons, including patient-related reasons (e.g., readmission to hospital, suffering from paroxysmal sympathetic hyperactivity) but also logistical reasons (e.g., discharge took place more quickly than expected and measurement could not be planned anymore). Ninety-seven patients had measurements on both admission and discharge.

### 3.2. Prevalence of Spasticity and Severe Spasticity

At EIN admission, EIN 88% (106 of 117 patients with valid data) had an AS ≥ 1 in any of the tested muscle groups. At discharge, this had increased to 90% (93 of 104 patients with valid data), with an AS ≥ 1 in any of the tested muscle groups (McNemar test, *p* = 0.774). Prevalence of spasticity in the upper limbs was 76% at admission and 82% at discharge (McNemar test, *p* = 0.814). For the lower limbs, prevalence of spasticity was 75% at admission and 81% at discharge (McNemar test, *p* = 0.677).

**Figure 2:**
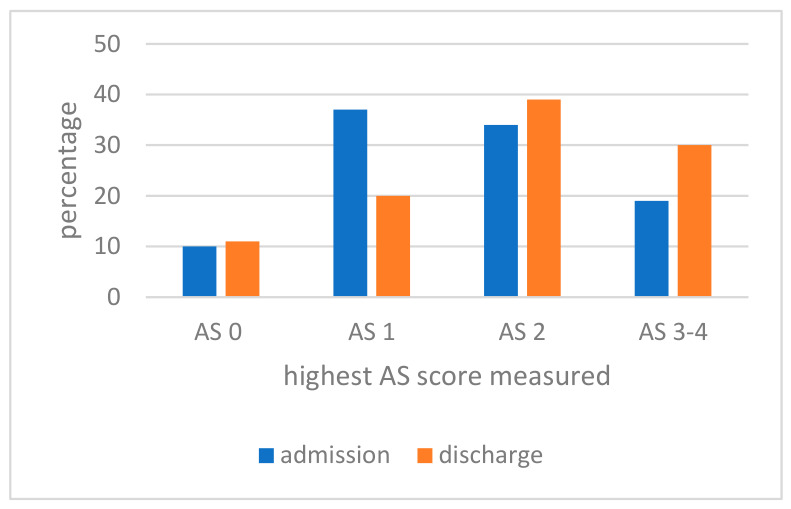
Distribution of the highest Ashworth Scale (AS) score.

Mean AS sum score at admission was 6.8 (SD 6.7; min–max 0–34); at discharge, mean AS sum score was 8.4 (SD 6.7; min–max 0–30) (Wilcoxon signed rank test, *p* = 0.172). Figure 2 shows the distribution of the highest AS score at admission and discharge. The percentage of patients with relatively low AS scores (i.e., AS scores 0 and 1) decreased towards discharge and the percentage with high AS increased.

At admission, 19% had an AS score of ≥3, and at discharge, 30% had an AS score of ≥3 (McNemar test, *p* = 0.123). For the upper limbs, this was 10% and 12% (McNemar test, *p* = 1.000), and for the lower limbs, 14% and 23% (McNemar test, *p* = 0.201).

### 3.3. Distribution of Spasticity

The overall prevalence of spasticity was 88%, yet the prevalence of spasticity in most individual muscle groups was less than 50%, as shown in Table 2, indicating a widespread distribution of spasticity.

The muscle groups most often affected were the elbow flexors (66%), followed by wrist flexors and hip adductors (both 50%) and knee flexors (49%). At discharge, all muscle groups, except knee extensors and plantar flexors, were affected in ≥50% of patients.

Looking at the distribution of spasticity, it was found that, at admission, 35% of patients was affected in all four limbs of the body, and at discharge, this had increased to 41%. At admission and discharge, only 4% had unilateral involvement of muscle groups. While not having spasticity in the upper limbs (only 24%), 56% and 44% still had spasticity in the lower limbs at admission and discharge from EIN.

### 3.4. Spasmolytics and Analgesics

The spasmolytics prescribed included baclofen orally and botulinum toxin injections. Four patients (3%) received botulinum toxin injections in the 3 months prior to admission, but exact timing is unknown. At admission, spasmolytics were prescribed in 32% of patients, which increased to 38% not significantly at discharge from EIN (McNemar test, *p* = 0.332). For patients with an AS score ≥3 at admission or discharge, this was 53% and 54%, respectively.

Analgesics prescribed included mainly paracetamol and NSAIDs. Occasionally, a patient was admitted with tramadol or morphine prescribed, but these were always discontinued during their stay. Overall, at admission, analgesics were prescribed to EIN 46% of patients; at discharge, this significantly decreased to 35% (McNemar test, *p* = 0.040). For patients with an AS score ≥3 at admission, this was 74% and 38% at admission and discharge, respectively.

A total of 17 of the 118 patients with valid data on both time points received botulinum toxin injections in the upper or lower limbs during EIN. All of these patients had a highest AS score of 3 or 4 at admission. No intrathecal baclofen pumps were implanted during EIN. Three intrathecal baclofen trials took place, two of which led to implantation later on. In three other patients, trialing was indicated but could not take place within the 14 weeks of EIN due to logistic reasons.

### 3.5. Factors Correlated with the Prevalence and Severity of Spasticity

At admission, only age showed only to be significantly correlated with having spasticity (OR 0.95 CI 0.92 to 0.99; *p* = 0.014), with having less often spasticity with the increase in age. At discharge, only the presence of spasticity at admission was associated with the presence of spasticity at the end of EIN (OR 9.257 CI 2.01 to 42.52; *p* = 0.004). Tables with OR and CI for all variables analyzed at admission and discharge can be found in Supplementary File S1.

**Figure 3:**
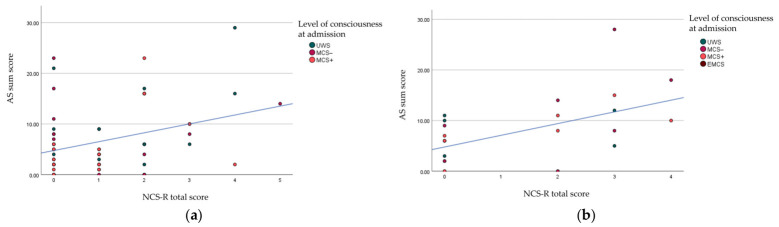
Scatterplot and fitted lines of NCS-R scores and AS sum scores (**a**) at admission; (**b**) at discharge. AS: Ashworth Scale; NCS-R: nociception coma scale-revised; UWS: Unresponsive Wakefulness Syndrome; MCS: Minimally Conscious State; EMCS: emerged from MCS.

At admission, there was a positive correlation between AS sum score and prescribed spasmolytics (*r* = 0.263; *p* = 0.016) and NCS-R scores (*r* = 0.334; *p* = 0.009). At discharge, there was a positive correlation between AS sum score at discharge and prescribed spasmolytics (*r* = 0.479; *p* < 0.001), longer TSI (*r* = 0.235; *p* = 0.041), higher NCS-R scores (*r* = 0.542; *p* = 0.008 (see Figure 3)), and higher AS scores at admission AS sum scores (*r* = 0.457; *p* < 0.001). Tables with the Pearson’s *r* for all variables analyzed can be found in Supplementary File S2.

### 3.6. Evolution of Spasticity and Correlated Factors

**Figure 4:**
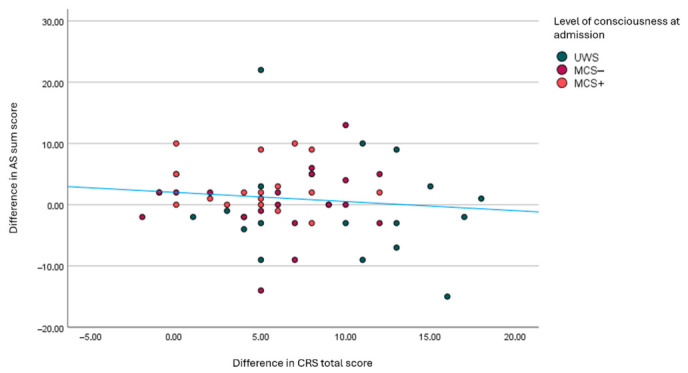
Scatterplot and fitted line of the difference in CRS-R scores and the difference in AS sum scores. CRS-R: coma recovery scale-revised; AS: Ashworth Scale; UWS: Unresponsive Wakefulness Syndrome; MCS: Minimally Conscious State.

To see how spasticity evolves over time, the difference in AS sum scores between discharge and admission was calculated. Of the 59 patients who had valid data for the difference in AS sum scores, the mean (SD) difference was 1.01 (6.1) with a range from –15 to 22. Figure 4 shows a scatterplot of the difference in CRS-R scores and the difference in AS sum scores. There was no correlation between the difference in CRS-R scores and the difference in AS sum scores (*r* = −0.114; *p* = 0.396). No correlations were found between other factors and AS sum scores, besides AS sum score at admission (*r* = −0.447; *p* < 0.001) (see Supplementary File S3).

**Figure 5:**
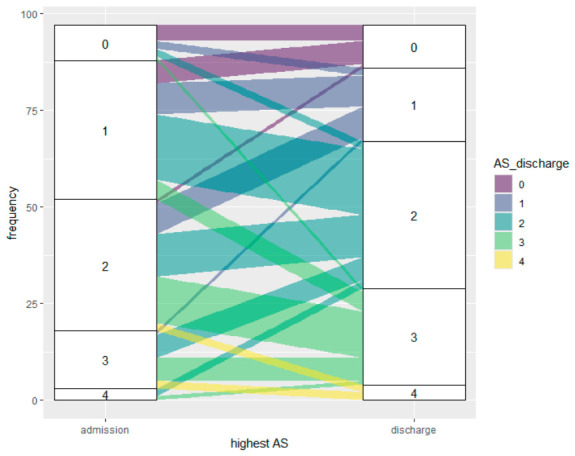
Evolution of the highest Ashworth Scale (AS) scores at admission and discharge.

Figure 5 shows the evolution of spasticity according to the highest AS in patients at admission. The highest AS score increased in 43 patients (44%); in 29 patients (30%), the highest AS score remained unchanged; and in 25 patients (26%), the highest AS score decreased. No significant relationship was found between LOC at admission and evolution of the highest AS score. Of the 38 patients in UWS at admission, 47% showed an increase in the highest AS. In contrast, 33% of the 33 patients in MCS− at admission (*n* = 11) and 54% of the 26 those in MCS+ (*n* = 14) showed an increase in the highest AS score. In patients who experienced an increase to AS 3 or 4, more muscle groups were involved at admission.

### 3.7. Evolution of Level of Consciousness and Spasticity

We analyzed whether there is a correlation between the presence and severity of spasticity at admission to EIN and the evolution of LOC. There was no correlation between having spasticity at admission and regaining consciousness yes/no during EIN (chi-squared test, *p* = 0.260) or the evolution of LOC (i.e., CRS-R at discharge minus CRS-R at admission).

**Figure 6:**
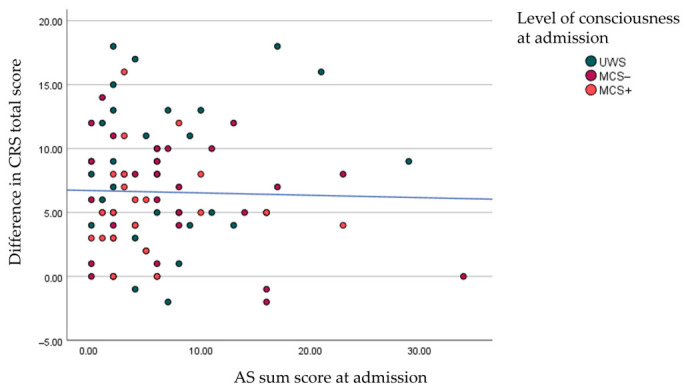
Scatterplot and fitted line of AS sum score at admission and difference in CRS-R total scores (i.e., CRS-R at discharge minus CRS-R at admission). AS: Ashworth Scale; CRS-R: coma recovery scale-revised; UWS: Unresponsive Wakefulness Syndrome; MCS: Minimally Conscious State.

There was no correlation between AS sum score at admission and regaining consciousness yes/no during EIN (*t*-test *p* = 0.269) or the evolution of LOC (*r* = −0.026; *p* 0.808) (see Figure 6).

## 4. Discussion

In this nationwide prospective longitudinal study involving a cohort of 126 PDOC patients, spasticity was present in 88% of patients at admission to PDOC specialized neurorehabilitation. This prevalence remained stable, with 90% at discharge. Spasticity was widespread, with only 4% unilateral involvement. Elbow flexors, wrist flexors, hip adductors, and knee flexors were affected most often. Only a proportion of patients received spasmolytics (36%) of analgesics (46%) at admission. Correlations between the AS sum score and the intake of spasmolytics, NCS-R, and TSI were found. The only predictor of spasticity at admission was age, while the only predictor at discharge was the severity of spasticity at admission. No significant relationship was found between the evolution of AS sum score and the evolution of LOC, neither between AS sum score at admission and the following evolution of LOC.

The high prevalence of 88% and 90% aligns with previous studies. The study by Zhang et al. (2021) [20] showed a prevalence of 95%, and the study of Thibaut et al. [11] 89%. The studies by Nakase-Richardson et al. [8] and Seel et al. [18] found a lower prevalence of 70%, and Ganesh et al. [7] found 57%. However, it is difficult to compare studies, as Nakase-Richardson et al. and Ganesh et al. reported only the presence or absence of spasticity and reported it as a complication. It is not clear if spasticity was measured using the (M)AS. In addition, most studies are retrospective and use data from medical records. Finally, the patients included in the different studies differs. For example, Thibaut et al. included patients with a mean TSI of 35 and 40 months. In our study, the mean TSI was 94 days (i.e., +/− 3 months).

In our cohort, 35% of patients had spasticity in all four limbs and 96% had bilateral involvement. Zhang et al. [20] reported the highest prevalence of spasticity in the internal shoulder rotators, followed by a high prevalence in the elbow flexors. In our study, elbow flexors and hip adductors appeared to be most often affected directly at admission, but shoulder internal rotators were not measured. Severe spasticity (i.e., AS ≥ 3) was present in 19% of patients at admission and in 30% of patients at discharge. Percentages at admission are much lower than those described by Zhang et al. [20], who reported 57%, but much higher than those described by Seel et al. [18], who reported percentages of 10%. However, the study settings and methodologies differed from the present study, reporting on cohorts with shorter TSI.

In our cohort only age seemed to be negatively associated with the presence of spasticity at admission. At discharge this correlation was absent. The Dutch medico-ethical context regarding end-of-life decision-making in PDOC could explain the relatively small number of older patients with severe spasticity in our sample [28]. It is possible that these individuals receive palliative care in hospital, instead of being referred to neurorehabilitation, resulting in a selection bias of older patients having relatively little spasticity.

In our cohort, a correlation was found between the AS sum score (i.e., severe spasticity or muscle involvement) and the NCS-R pain/discomfort score, both at admission and discharge. The relationship between the AS sum score and the NCS-R aligns with the findings of Thibaut et al. [11] in a more chronic population and suggests a potential bidirectional interaction, in which spasticity may cause discomfort that can have a non-specific effect on the testing of consciousness or, conversely, discomfort may exacerbate spasticity. But the NCS-R scores in our cohort, which were measured when performing the CRS-R, were very low, which appears to attenuate the role of pain as a contributing factor. The use of spasmolytics and analgesics (at discharge) was also correlated with a higher AS sum score. The relationship between the intake of spasmolytics and the AS sum score is to be expected, but also raises the question about the efficacy of oral medication. It should be noted that no dose–effect relationship was studied and no conclusions about causality can be drawn.

Regarding other clinical factors that could predict the severity of spasticity, Thibaut et al. [11] found correlations between TSI and the presence of spasticity, with patients measured at longer TSI having more often spasticity. However, the population was in chronic PDOC lasting for 3 years on average. In addition, Winters et al. [19] found that etiology also influenced the evolution of spasticity. Other factors were probably not found because of the homogeneous population.

The relationship between spasticity and recovery of consciousness is complex and possibly bidirectional. Spasticity may worsen as patients regain responsiveness to nociceptive stimuli, but spasticity may also improve alongside improvements of consciousness as both reflect underlying neurological recovery [13,14]. However, our study did not provide clear evidence supporting either of these hypothesis as a consistent or generalizable trend. These findings suggest that changes in spasticity do not necessarily parallel recovery of consciousness. Therefore, spasticity may need to be considered and managed as a partly independent clinical feature in this population. Further research is warranted to clarify this relationship. Yet, there may be conflicting demands between spasticity management and the promotion of consciousness. For example, oral baclofen, an important spasmolytic, is also sedating, with possible negative effects on level of consciousness.

No association was found between the presence of severe spasticity at admission to EIN and a lack of recovery of level of consciousness (LOC) during the short period of EIN. However, it has been hypothesized that spasticity may interfere with the assessment of consciousness by negatively affecting motor responses [11].

Although this study is the first to investigate prospectively spasticity in virtually all newly diagnosed PDOC patients in one country, it is not without limitations.

In this study, AS was used as a measure of spasticity; however, the AS also assesses other characteristics beyond the velocity-dependent increase in muscle tone [23]. This lack of specificity warrants caution interpretating results of spasticity in a strict neurophysiological sense. Future research should include for example the Tardieu scale, which is also able address contractures and demonstrated good inter- and intra-rater reliability [29]. Nonetheless, despite its significant limitations, the AS remains a practical scale and a useful bedside screening tool for patients who may lack voluntary movement. Furthermore, tone abnormalities in patients with PDOC have been often labeled as spasticity in the literature. However, clinical observations frequently reveal patterns of “gegenhalten”, which is more consistent with paratonia than with spastic dystonia [30]. It is crucial to further delineate the various clinical manifestations of increased tone before identifying appropriate treatment strategies [13,14,31].

Level of consciousness was assessed using the CRS-R, which is the golden standard. However, responses do require motor abilities, which may be affected by spasticity. When evaluating the relationship between spasticity and level of consciousness in future research, other additional methods for the evaluation of level of consciousness should be considered.

The time frame of our study was 14 weeks maximally, which is the maximum duration of EIN. This is a relatively short timeframe to assess the evolution of spasticity. This brief period of intensive neurorehabilitation, focused on optimal stimulation, also explains why more invasive procedures such as surgery or intrathecal baclofen (ITB) implantation were often postponed until after the study period. Finally, this study was not designed to evaluate several important research questions that are of importance when discussing spasticity in PDOC, e.g., (1) the effects of specific treatments could not be evaluated; there was no systematic collection of information concerning dosing of medication and the effect on spasticity; (2) the impact of other comorbidities (e.g., paroxysmal sympathetic hyperactivity and heterotopic ossification) on the presence and/or development of spasticity; (3) the role of spasticity in contracture development; and (4) the assessment of spasticity withing the ICF framework to evaluate the impact of spasticity on daily activities (performed by caregivers), like sitting on a wheelchair or washing and dressing.

## 5. Conclusions

This study is the first to prospectively investigate spasticity in almost all newly diagnosed PDOC patients in one country through the same EIN program. This protocol provides valuable insights into the prevalence, distribution, and evolution of spasticity in patients with PDOC. The findings underline several important conclusions, as follows.

- The study confirmed a high prevalence of spasticity in the PDOC population, with rates of 88% at admission and 90% at discharge. The distribution pattern throughout different muscle groups is widespread with bilateral involvement in almost all patients.
- This study could not confirm circulating hypotheses concerning the evolution of level of consciousness in relation to the evolution of spasticity, nor could it confirm that spasticity can be considered as a poor prognostic factor for the evolution of LOC on the short term.
- The relationship between increased spasticity and the presence of potential pain (NCS-R scores) was identified without establishing the direction of the relationship.
- Even during specialized neurorehabilitation, prevalence and severity of spasticity increased over the 14-week follow-up period and the use of spasmolytics was associated with higher AS sum scores which questions the efficacy of oral treatment.

This study highlights the need for prospective interventional studies to optimize pharmacological and non-pharmacological spasticity management in patients with PDOC. Moreover, elucidating the role of spasticity in contracture development is essential to improve outcomes and quality of life in PDOC patients.

## Figures and Tables

**Table 1:** Clinical and demographic characteristics.

Characteristics	N = 126
Age *mean* (*SD*)	38.3 (16.5)
Gender *N* (%) male female	65 (52%) 61 (48%)
Type of injury *N* (%) traumatic brain injury non-traumatic brain injury	71 (56%) 55 (44%)
Level of consciousness at admission *N* (%) UWS MCS− MCS+	47 (37%) 45 (36%) 34 (27%)
Level of consciousness at discharge *N* (%) UWS MCS− MCS+ EMCS Deceased	11 (9%) 12 (10%) 42 (33%) 55 (44%) 6 (5%)
Time since injury at admission in days *mean* (*SD*)	71.0 (28.1)
Received spasmolytic medication at admission *N* (%)	36 (32%)
Received analgesic medication at admission *N* (%)	52 (46%)
Length of stay EIN *mean* (*SD*)	94.3 (37.2)
Pain (NCS-R) at admission *median*	1

N: number; SD: standard deviation; UWS: Unresponsive Wakefulness Syndrome; MCS: Minimally Conscious state; EMCS: emerged from minimally conscious state; EIN: early intensive neurorehabilitation; NCS-R: nociception coma scale-revised.

**Table 2:** (**a**) Distribution of severity of spasticity per muscle group and the highest Ashworth Scale (AS) at admission. (**b**) Distribution of severity of spasticity per muscle group and the highest Ashworth Scale (AS) at discharge.

(**a**)				
	**AS 0**	**AS 1**	**AS 2**	**AS 3 or 4**
Elbow flexors	52 (44%)	47 (40%)	16 (13%)	4 (3%)
Elbow extensors	74 (62%)	29 (24%)	10 (8%)	6 (5%)
Wrist flexors	60 (50%)	38 (32%)	14 (12%)	8 (7%)
Hip adductors	58 (50%)	38 (33%)	15 (13%)	5 (4%)
Knee flexors	60 (51%)	34 (29%)	14 (12%)	9 (8%)
Knee extensors	85 (73%)	21 (18%)	7 (6%)	4 (3%)
Plantar flexors	69 (63%)	28 (26%)	6 (6%)	7 (6%)
(**b**)				
	**AS 0**	**AS 1**	**AS 2**	**AS 3 or 4**
Elbow flexors	32 (31%)	35 (34%)	26 (26%)	9 (9%)
Elbow extensors	50 (49%)	38 (27%)	10 (10%)	4 (4%)
Wrist flexors	41 (40%)	34 (33%)	19 (18%)	9 (9%)
Hip adductors	38 (36%)	34 (32%)	24 (23%)	9 (9%)
Knee flexors	39 (37%)	32 (31%)	26 (25%)	8 (8%)
Knee extensor	67 (64%)	20 (19%)	12 (11%)	6 (6%)
Plantar flexors	58 (60%)	24 (25%)	7 (7%)	5 (5%)

## Data Availability

The datasets that support the findings of this ongoing study are not yet publicly available but are available from the corresponding author upon reasonable request.

## Supplementary Materials

The following supporting information can be downloaded at: https://www.mdpi.com/article/10.3390/brainsci16050524/s1, Supplementary File S1: predictors of spasticity at admission and discharge; Supplementary File S2: correlations between AS sum scores and clinical and demographic factors at admission and discharge; Supplementary File S3: Correlations between the difference in AS sum score and clinical and demographic factors.

## Abbreviations

The following abbreviations are used in this manuscript:

AS	Ashworth Scale
CI	Confidence interval
CRS-R	Coma Recovery Scale-Revised
DOC	Disorders of Consciousness
DOCTOR	Disorders Of Consciousness, Treatment and Outcomes Registry
EIN	Early Intensive Neurorehabilitation
EMCS	Emerged from MCS
LOC	Level of Consciousness
LoS	Length of Stay
MAS	Modified Ashworth Scale
MCS	Minimally Conscious State
NCS-R	Nociception Coma Scale-Revised
OR	Odds Ratio
PDOC	Prolonged Disorders of Consciousness
TBI	Traumatic Brain Injury
TSI	Time Since Injury
UWS	Unresponsive Wakefulness Syndrome
