# Supplementary material for: Spasticity in Prolonged Disorders of Consciousness: A Prospective Cohort Study
Source: Brain Sci. 2026 May 14;16(5):524. doi: 10.3390/brainsci16050524 (PMC13204021; doi:10.3390/brainsci16050524)
Supplement: Supplementary file 1 [file brainsci-16-00524-s001.zip › brainsci-4289191-supplementary.pdf]

Supplementary file S1.

predictors of spasticity at admission

	Odds Ratio and Confidence interval	P value
Age	.95 (.92 to .99)	.014*
Gender		
male	1	
female	.63 (.19 to 2.10)	.446
Type of injury		
non-traumatic brain injury	1	
traumatic brain injury	.95 (.28 to 3.20)	.937
Level of consciousness at admission– CRS-R total score	1.01 (.87 to 1.16)	.930
Level of consciousness at admission		.086
UWS	1	
MCS-	.30 (.08 to 1.23)	.096
MCS+	.54 (.205 to 20.91)	.537
Time since injury at admission in days	1.01 (.98 to 1.03)	.639
Spasmolytic medication at admission		
No	1	
Yes	**	.998
Analgesic medication at admission		
No	1	
Yes	.205 (.50 to 8.43)	.318
Pain/discomfort (NCS-R) at admission	1.20 (.65 to 2.19)	.576

* significant at p <.05; ** high OR, but not significant therefore no odds ratio and confidence interval
CRS-R: coma recovery scale-revised; UWS: unresponsive wakefulness syndrome; MCS: minimally conscious state; NCS-R: nociception coma scale-revised

Predictors of spasticity at discharge

	Odds Ratio and Confidence interval	P value
Age	.97 (.93 to 1.01)	.143
Gender		
male	1	
female	.75 (.21 to 2.62)	.651
Type of injury		
non-traumatic brain injury	1	
traumatic brain injury	1.26 (.36 to 4.44)	.717
Level of consciousness at admission – CRS-R total score	1.01 (.87 to 1.16)	.946
Level of consciousness at discharge – CRS total-R score	1.03 (.917 to 1.17)	.588
Level of consciousness at discharge		.648
UWS	1	
MCS-	**	.999
MCS+	.57 (.06 to 5.41)	.628
EMCS	1.48 (.14 to 15.94)	.746
Time since injury at admission in days	1.01 (.99 to 1.04)	.279
Spasmolytic medication at admission		
No	1	
Yes	4.91 (.60 to 40.35)	.139

Spasmolytic medication at discharge		
No	1	
Yes	7.00 (.85 to 57.89)	.071
Analgesic medication at admission		
No	1	
Yes	1.19 (.29 to 4.97)	.813
Analgesic medication at discharge		
No	1	
Yes	4.50 (.916 to 22.11)	.064
Length of stay EIN in days	1.02 (.99 to 1.04)	.094
Nociception Coma Scale-revised admission	1.11 9.54 to 2.27)	.779
Nociception Coma Scale-revised discharge	1.58 (.71 to 3.49)	.262
Spasticity at admission	9.26 (2.02 to 42.53)	.004*

* significant at p <.05; ** high OR, but not significant therefore no odds ratio and confidence interval

CRS-R: coma recovery scale-revised; UWS: unresponsive wakefulness syndrome; MCS: minimally conscious state; EMCS: emerged from minimally conscious state; EIN: early intensive neurorehabilitation; NCS-R: nociception coma scale-revised

Supplementary file S2.

Correlations between AS sum score at admission and clinical and demographic factors

	Pearson's *r*	P value
Age	-.121	.243
Gender	-.016	.879
Type of injury	-.151	.145
Level of consciousness at admission – CRS total score	-.019	.852
CRS-R motor subscale	.034	.761
Time since injury at admission in days	-.065	.529
Spasmolytic medication at admission	.263	.016*
Analgesic medication at admission	.156	.157
Pain/discomfort at admission – NCS-R	.334	.009**
* significant at p <.05; when prescribed spasmolytics, higher AS sum scores; ** significant at p<.05<br>CRS-R: coma recovery scale-revised; NCS-R: nociception coma scale-revised		

Correlations AS sum score at discharge and clinical and demographic factors

	Pearson's *r*	P value
Age	-.221	.055
Gender	.100	.390
Type of injury	-.045	.696
Level of consciousness at admission – CRS total score	-.052	.657
CRS-R motor subscale at admission	.040	.745
Level of consciousness at discharge – CRS total score	-.067	.572
CRS-R motor subscale at discharge	-.084	.666
Time since injury at admission in days	.235	.041**
Spasmolytic medication at admission	.042	<.001*
Analgesic medication at admission	.239	.046**
Spasmolytic medication at discharge	.479	<.001*
Analgesic medication at discharge	.048	.703
Pain/discomfort at admission – NCS-R	.150	.280
Pain/discomfort at discharge – NCS-R	.542	.008**
AS sum score at admission	.457	<.001**
* significant at p <.05; when prescribed spasmolytics/analgesics, higher AS sum scores; ** significant at p<.05;<br>CRS-R: coma recovery scale-revised; NCS-R: nociception coma scale-revised; AS: Ashworth Scale		

Supplementary file S3.

Correlations between the difference in AS sum score and clinical and demographic factors

	Pearson's *r*	P value
Age	-.105	.428
Gender	.181	.169
Type of injury	.042	.749
Level of consciousness at admission – CRS total score	.093	.482
Difference in CRS-R scores	-.114	.396
CRS-R motor subscale at admission	.037	.794
Difference in CRS-R motor subscale	-.357	.112
Time since injury at admission in days	.107	.419
Spasmolytic medication at admission	.067	.632
Analgesic medication at admission	.031	.823
Nociception Coma Scale-revised admission	.011	.942
Difference in NCS-R score	.345	.227
AS sum score at admission	-.447	<.001

* significant at p <.05; when prescribed spasmolytics/analgesics, higher AS sum scores

** significant at p<.05
